# A Novel Endogenous Indole Protects Rodent Mitochondria and Extends Rotifer Lifespan

**DOI:** 10.1371/journal.pone.0010206

**Published:** 2010-04-21

**Authors:** Burkhard Poeggeler, Kumar Sambamurti, Sandra L. Siedlak, George Perry, Mark A. Smith, Miguel A. Pappolla

**Affiliations:** 1 Department of Dermatology, University of Luebeck, Luebeck, Germany; 2 Department of Neurosciences, Medical University of South Carolina, Charleston, South Carolina, United States of America; 3 UTSA Neurosciences Institute and Department of Biology, College of Sciences, University of Texas at San Antonio, San Antonio, Texas, United States of America; 4 Department of Pathology, Case Western Reserve University, Cleveland, Ohio, United States of America; Roswell Park Cancer Institute, United States of America

## Abstract

Aging is a multi-factorial process, however, it is generally accepted that reactive oxygen species (ROS) are significant contributors. Mitochondria are important players in the aging process because they produce most of the cellular ROS. Despite the strength of the free-radical hypothesis, the use of free radical scavengers to delay aging has generated mixed results in vertebrate models, and clinical evidence of efficacy is lacking. This is in part due to the production of pro-oxidant metabolites by many antioxidants while scavenging ROS, which counteract their potentially beneficial effects. As such, a more effective approach is to enhance mitochondrial metabolism by reducing electron leakage with attendant *reduction* of ROS generation. Here, we report on the actions of a novel endogenous indole derivative, indolepropionamide (IPAM), which is similar in structure to melatonin. Our results suggest that IPAM binds to the rate-limiting component of oxidative phosphorylation in complex I of the respiratory chain and acts as a stabilizer of energy metabolism, thereby reducing ROS production. IPAM reversed the age-dependent decline of mitochondrial energetic capacity and increased rotifer lifespan, and it may, in fact, constitute a novel endogenous anti-aging substance of physiological importance.

## Introduction

The reasons for the failure of antioxidant therapies in AD may be multiple, but toxic, pro-oxidant intermediates generated from many such compounds can reverse their beneficial effects and have been demonstrated to be partly responsible for the failure [1]. Consequently, administration of reactive antioxidants like vitamin E have resulted in unexpectedly unfavorable clinical outcomes [2]. Another frequent reason for failure is lack of “on-site protection”, a consequence of limited bioavailability.

Despite the strength of the free-radical hypothesis, the use of free radical scavengers to delay aging has generated mixed results in vertebrate models, and clinical evidence of efficacy is lacking [3]. We previously showed that this may be due, in part, to the production of pro-oxidant metabolites by many antioxidants while scavenging ROS, which counteract their potentially beneficial effects [1]. As such, a more effective approach is to enhance mitochondrial metabolism by reducing electron leakage with attendant *reduction* of ROS generation [4].

Mitochondria are important players in the aging process because they produce most of the cellular reactive oxygen species (ROS) [5]–[7]. These organelles are the main source of most free-radicals generated by cells and defects in energy metabolism have implicated these organelles in various neurodegenerative diseases and in aging [8], [9]. It has been proposed that in aging, a vicious cycle can occur in which increased oxidative stress leads to persistent mitochondrial dysfunction and severe energy depletion [9], [10].

We have previously hypothesized that such damage can either be attenuated or reversed by compounds that act as mobile electron and proton carriers [11]. Some indole substances are capable of supporting electron flux through the respiratory chain, preventing the breakdown of the mitochondrial membrane potential, and decreasing electron leakage, and they thereby reduce the formation of free-radicals [12]. In addition, certain indoles may be potent anti-oxidants via their involvement in single-electron transfer reactions that lack pro-oxidant intermediates [8], [12]. In 1999, we reported that a substance related to melatonin, indole-3-propionic acid (IPA) may be the most potent naturally occurring hydroxyl radical scavenger in neutralizing free-radicals by electron donation [1]. However, IPA is hydrophilic and has limited bioavailability, which raises doubts as to the physiological relevance of this molecule. In order to overcome this problem, we designed, by introducing an amide group to IPA, an amphiphilic indole substance (indolepropionamide; IPAM) which has a higher bioavailability. After synthesizing IPAM, we discovered that it existed as a naturally occurring substance. Further, because we reported that certain indoles neutralized free radicals by electron donation, we investigated the effect of IPAM on the mitochondrial membrane potential, the proton motive force that drives ATP synthesis, in preparations from rat brain of young and old animals. We also tested whether IPAM could prevent and protect against the deleterious effects of specific mitochondrial toxins.

## Materials and Methods

### High performance liquid chromatography (HPLC)

HPLC with fluorometric detection was carried out on supernatants of rat brain samples that were homogenized in 0.4 N perchloric acid (PCA) including as additives 0.1% EDTA and 0.05% Na_2_S_2_O_5_. The mobile phase consisted of 20% methanol, 30 mM NaH_2_PO_4_, 50 mM citric acid, 2 mM octanesulfonic acid and 0.1 mM EDTA at a flow rate of 0.5 ml/minute.

### Synthesis of IPAM

IPAM had initially been produced as follows: a mixture of 30 g of indole-3-propionic acid and 10 ml of methanesulfonic acid in 200 ml of ethanol was stirred for 24 hours, poured into water, and extracted with ethylacetate. The ethylacetate solution was washed with NaHCO_3_ solution and water and dried over magnesium sulfate. A solution of 800 mg of the crude product (indole-3-propionic acid ethyl ester) and 2 ml of hydrazine in 20 ml of ethanol was refluxed for 18 hours, and extracted with ethylacetate. The organic phase was washed with brine, dried over magnesium sulfate, and evaporated at reduced pressure to give with 93% yield the intermediate propanoic acid hydrazide as a solid. This material and 0.3 g of Raney nickel catalyst (W-4) in 25 ml of ethanol were refluxed for 2.5 hours. The solution was decanted and evaporated at reduced pressure and the residue chromatographed on silicia gel, eluting with ethylacetate, to give with 96% yield indole-3-propionamide.

### Mitochondrial preparations and measurement of membrane potentials

Mitochondria were prepared and maintained as described in detail [13]. Mitochondrial membrane potential was determined by measuring rhodamine 123 fluorescence quenching after respiration-driven uptake of the dye by mitochondria of the rat brain preparations in 6 mM malate and 6 mM glutamate for 30 minutes. Rhodamine fluorescence was monitored in real time as previously described [14]. The negative mitochondrial membrane potential was calculated by using the Nernst-Guggenheim equation: Δ ψ  = 59 log ([rhodamine]_in_/[rhodamine]_ out_), according to Scaduto and Grotyohann [15].

To examine potential interactions between IPAM and mitochondrial complexes, sub-mitochondrial preparations were obtained according to published methods [16] to measure the specific activity of mitochondrial oxidative complexes, where intact mitochondria were harvested and maintained as described in detail [13]. The protocols of Durand et al. [17] were used for all incubation conditions to determine the effects of IPAM on energy metabolism using mitochondria or sub-mitochondrial particles. In addition, IPAM binding to complex I was determined by displacement of known ligand inhibitors to this complex. For this purpose, the methods of Brenner-Lavie et al. [18] and Yuan and Pang [19] were employed to determine [^3^H]-dopamine and 2-[^125^I]-iodomelatonin displacement by IPAM over a large concentration range.

The specific activity of the mitochondrial oxidative complexes was determined using sensitive assays as described by Martin et al. [16], [20] at an incubation temperature of 37.5°C. Mice brain mitochondria preparations from young and old animals (male Swiss Webster mice, n = 6) were used, demonstrating again the effects of aging, melatonin and IPAM at concentrations of 10 nM. Sub-mitochondrial particles from mouse brain were incubated with a solution containing nitroblue tetrazolium (NBT) at 0.1% in working buffer for 30 minutes at an incubation temperature of 37.5°C in an oscillating water bath. The NBT reduction assay [21] was carried out in the presence of 100 Units of superoxide dismutase (SOD) to prevent non-specific reduction of the tetrazolium dye by superoxide anion radicals generated during the incubation. Control experiments with the specific complex I inhibitors capsaicin and dopamine as described above were carried out to assure that more than 80% of NBT reduction was due to specific reduction of the dye as an alternate electron and proton acceptor at the mitochondrial iron sulfur cluster N2 in complex I. Termination of the assay and extraction of the reduced dye was carried out by centrifugation of the mitochondrial preparations at 2000 g for 10 minutes. The supernatant was decanted and the pellet was resuspended in 1 ml glacial acetic acid. The relative absorbance of the glacial acetic acid fraction was measured at 570 nm to calculate the amount of reduced diformazan formed during the incubation by reduction of the tetrazolium dye using a standard curve generated with synthetic nitroblue diformazan.

### Antioxidant and pro-oxidant activity of IPAM and related indoles

The test compounds and salicylate were incubated at a concentration of 0.1 mM in the presence of 1 mM hydrogen peroxide, 0.1 mM FeCl_3_ and 1 mM EDTA. The hydroxyl radical adducts of salicylate, 2,3-DHBA, and 2,5-DHBA were measured by HPLC-ECD. The measurements are expressed as a percentage of control and represent the means ± SEM from 6 independent experiments.

Inhibition of hydroxyl radical mediated oxidative damage by IPAM compared with related indole antioxidants in rat forebrain homogenate shows that IPAM was very powerful preventing OH• mediated DNA damage. Rat forebrain homogenate was incubated for sixty minutes with 3 mM hydrogen peroxide, 4 mM ferrous sulfate, and 2 mM ADP to generate hydroxyl radicals. The IC_50_ values (concentrations of each indole compound required to reduce oxidative DNA damage by 50%) are given as means + standard deviations for N = 6 different determinations. The IC_50_ values were calculated by performing six experiments each at increasing concentrations ranging from 0.01 to 100 µM melatonin, indole-3-propionic acid, and IPAM. DNA damage was examined by measuring the formation of 8-hydroxydeoxyguanosine by a sensitive HPLC method applying electrochemical detection in the presence and absence of the hydroxyl radical scavengers.

### Animal experiments

Sprague-Dawley rats, aged 1 month (n = 6) and 20 months (n = 6) were maintained according to the approved institutional protocols. Upon sacrifice, animals were euthanized and brains removed. Mitochondria were isolated as previously described (13). Mitochondria were also prepared from young (3 month, n = 6) and old (18 months, n = 6) male Swiss Webster mice, which were maintained using approved protocols. All animals were strictly handled and sacrificed according to humane protocols reviewed and approved by the MUSC Institutional Animal Care & Use Committee, which is part of the MUSC Office of Research Integrity.

### Rotifer experiments

The rotifers were housed in individual isolation cultures of defined and uniform age as described previously in detail [11].

## Results

**Figure 1:**
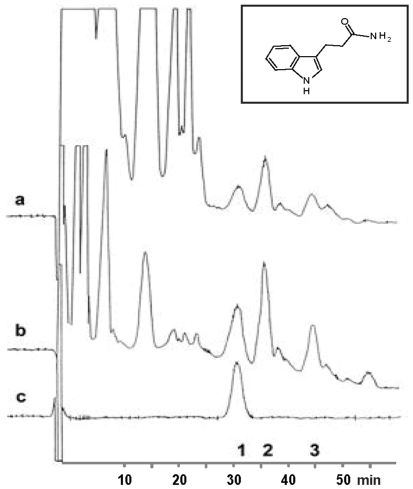
Fluorometric HPLC detection of IPAM, melatonin and IPA in the brain of one-month-old male Sprague-Dawley rats: Peaks with identical elution times as synthetic IPAM, melatonin and IPA were consistently observed in rat brain. a) One hour after administration of 300 mg/kg L-tryptophan, IPAM (1), melatonin (2) and indole-3-propionic acid (3) concentrations reached levels of 346±9 (IPAM), 713±32 (MEL) and 281±14 (IPA) pg indole/mg protein, respectively, in rat brain (n = 6). b) Co-elution of the endogenous indoles IPAM, melatonin and IPA along with the standards of these indole compounds results in larger single elution peaks representing endogenous plus exogenous indoles. c) Synthetic IPAM standard (1 ng) eluted at 30 minutes. Inset shows predicted structure of synthetic IPAM.

We designed, by introducing an amide group to IPA, an amphiphilic indole substance (IPAM), and its predicted structure is shown in Figure 1, inset. After synthesizing IPAM, we discovered that it existed as a naturally occurring substance. Endogenous IPAM (Figure 1a). eluted with an identical retention time as synthetic IPAM (Figure 1c) by HPLC Initially, peaks corresponding to endogenous IPAM were inconsistent and could not be accurately quantified due to their very low basal concentrations which, in rat brain tissue were estimated to be below 100 pg indole/mg protein. In order to increase the sensitivity of our method and to avoid potential artifacts, we pursued an established strategy for indole detection consisting of administering an oral load of tryptophan, the aromatic amino-acid precursor of indoles. To further confirm the observation in 1a, synthetic IPAM, melatonin and IPA (1 ng of each compound/mg protein) were added to tissue samples prepared in parallel (containing the endogenous indoles in the tissue at levels as depicted in Figure 1a) and the samples were analyzed by HPLC (Figure 1b). One hour after administration of 300 mg/kg L-tryptophan to 1 month old male Sprague-Dawley rats, we observed that IPAM, melatonin and indole-3-propionic acid concentrations could be reproducibly measured as shown in Figure 1 at increased levels of 346±9 (IPAM), 713±32 (MEL) and 281±14 (IPA) pg indole/mg protein in rat brain (n = 6).

To determine the bioavailability of each substance, brain samples analyzed at 2, 4 and 8 hours after intraperitoneal administration of synthetic IPAM (n = 6), melatonin (n = 6) or IPA (n = 6) to 1 month old male Sprague-Dawley rats. Intraperitoneal administration of 0.5 mg/kg melatonin or 0.5 mg/kg IPA did not increase the barely-detectable baseline levels of these indoles in rat brain. In contrast, IPAM given at the same dose resulted in high brain levels of IPAM of 691±23 at 2 hours, 562±13 at 4 hours and 361±12 pg indole/mg protein at 8 hours (n = 6, mean ± SEM).

**Figure 2:**
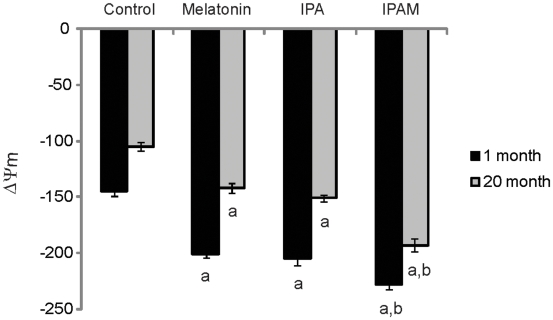
Mitochondrial membrane potential in mV: Effects of age and indole agents. Mitochondrial membrane potential in mV (mean ± SEM) measured in rat brain mitochondria from young and old animals (male Sprague-Dawley rats, n = 6) with effects of melatonin, IPA and IPAM at 10 nM. All compounds were significantly different from control at both 1 month and 20 months, with IPAM showing significantly greater effects than melatonin or IPA. a- significantly different from control (p<0.0005); b- significantly different from melatonin and IPA (p<0.01).

The effect of age and added indole agents on mitochondrial membrane potential was measured. A pronounced age-dependent decline in proton motive force and energetic capacity of untreated mitochondria was seen. Notably, these age-related effects were antagonized by administration of IPAM, melatonin and IPA (Figure 2).

**Figure 3:**
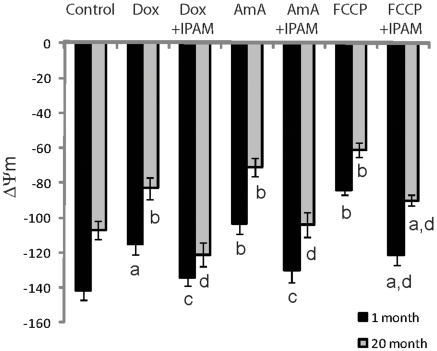
Mitochondrial membrane potential in mV: Effects of age, toxins and IPAM. Mitochondrial membrane potential in mV (mean ± SEM) was measured in rat brain mitochondria from young and old animals (male Sprague-Dawley rats, N = 6). The toxins doxorubicin (DOX), antimycin A (AmA) and FCCP at concentrations of 500 nM led to significant reductions in membrane potential as compared to control. Co-administration of IPAM at 10 nM significantly antagonized the effects of each toxin. In fact, the toxicity of doxorubicin and antimycin A was completely abrogated by IPAM to levels comparable to control. a- significantly different from control (p<0.05); b-significantly different from control (p<0.001); c- significantly different from toxin only (p<0.05); d-significantly different from toxin only (p<0.005).

The effectiveness of IPAM to prevent and protect against the deleterious effects of specific mitochondrial toxins in doxorubicin, antimycin A employed to inhibit electron transport, and carbonylcyanide-*p*-trifluoromethoxyphenylhydrazone (FCCP) to dissipate the proton potential, was determined. All three mitochondrial toxins, including FCCP, were able to induce a collapse of the mitochondrial proton potential. IPAM markedly reduced the proton potential collapse induced by the mitochondrial toxins to nearly baseline levels in both young and old rats (Figure 3).

**Figure 4:**
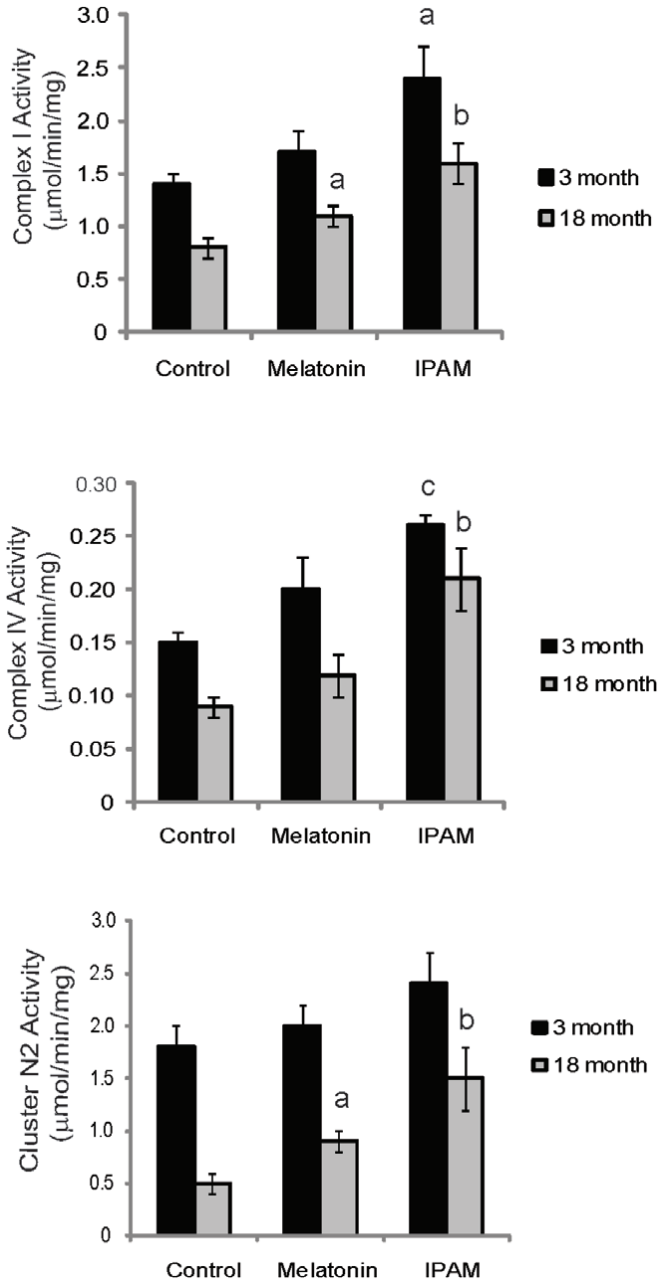
Activity of mitochondrial complex I and IV: Effects of age and indole agents on ferric cyanide reduction and cytochrome c oxidation. The activities of mitochondrial complex I (A) and complex IV (B) expressed in µmol/min/mg protein (mean ± SEM) were measured in mice brain mitochondrial preparations from young and old animals (male Swiss Webster mice, n = 10). Melatonin and IPAM were used at 10 nM concentration. Only IPAM consistently significantly increased complex I and IV activities compared to control. The results for complex I were verified by a second method in which the activity of the mitochondrial iron sulfur cluster N2 in complex I was determined by the nitroblue tetrazolium (NBT) reduction assay as µmol diformazan formed/minute/mg of mitochondrial protein (mean ± SEM) (C). a-significantly different from control (p<0.05); b-significantly different from control (p<0.005); c- significantly different from control (p<0.001).

IPAM, and melatonin to a lesser extent, can increase complex I and complex IV activity in the mitochondrial electron transport chain (Figures 4A,B). The results for complex I were verified by a second method in which the activity of the mitochondrial iron sulfur cluster N2 in complex I was determined by the NBT reduction assay as µmol diformazan formed/minute/mg of mitochondrial protein (mean ± SEM) (Figure 4C). No significant changes were seen in the activity of complex II to III (data not shown). Highly efficient and specific displacements of both ligands from their endogenous mitochondrial binding sites were also demonstrated (data not shown).

**Figure 5:**
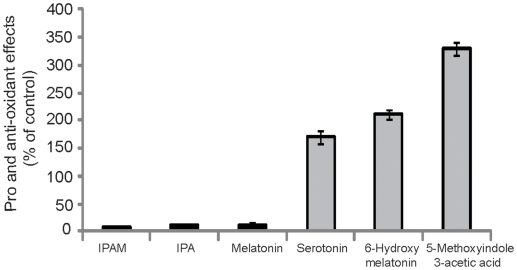
Pro- and antioxidant effects of indole agents expressed as hydroxyl radical adducts of salicylate (percentage of control, no test agent added). Pro- and antioxidant effects of indole agents as demonstrated as percentage of hydroxyl radical adducts formed from salicylate oxidation to 2,3- and 2,5-dihydroxybenzoic acids (DHBAs) versus control (incubation system without test agents: 100±2.8%, mean ± SEM, n = 6).

To assay for anti- and pro-oxidant activity, a hydroxyl radical-generating system consisting of hydrogen peroxide, iron, and EDTA was employed as described previously [1]. IPAM was the most potent antioxidant compound in reducing the formation of hydroxyl radicals and like IPA, it did not elicit any pro-oxidant reactive intermediates *in vitro* (Figure 5). On the other hand, closely related indole compounds such as the melatonin precursor serotonin and the melatonin metabolite 6-hydroxymelatonin, as well as the indolepropionic acid analogue 5-methoxyindoleacetic acid used as controls in the experiments, were also pro-oxidant and increased hydroxyl radical formation in this assay system. Inhibition of hydroxyl radical mediated oxidative damage by IPAM compared with related indole antioxidants in rat forebrain homogenate shows that IPAM attenuates OH• mediated DNA damage and the formation of 8-hydroxydeoxyguanosine. The IC_50_ values (concentrations of each indole compound required to reduce oxidative DNA damage by 50%) were determined to be 1.4±0.16 µM for melatonin, 7.46±0.80 µM for indole-3-propionic acid, and 0.18±0.03 µM for IPAM.

**Figure 6:**
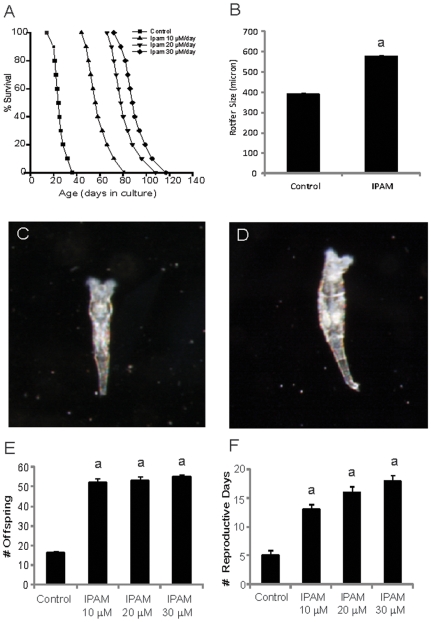
Lifespan extension in rotifers. In rotifers (n = 11) IPAM at 10, 20 and 30 µM markedly extended the lifespan of rotifers. These effects were dose dependent and highly significant at all concentrations tested (p values <0.001 for all concentrations versus the control group (A). IPAM treatment of rotifers for 15 days at 30 µM daily resulted in a significantly increased size (B). Representative size differences are illustrated in animals treated either with vehicle for 15 days (C) or IPAM (D) for 15 days. Magnification X 90. The number of offspring for individual parents (E), as well as the number of reproductive days (F) during lifetime was significantly increased with IPAM (n = 10). a- significantly different from control (p<0.0001).

We then used IPAM to test the hypothesis that free radicals may contribute to the aging process in a rotifer model of aging. For these experiments, we used the Bdelloid rotifer Philodina acuticornis odiosa Milne. IPAM was used at concentrations of 10, 20 and 30 µM (Figure 6A). Administration of IPAM resulted in significant life extension in the rotifers, even exceeding 300% at the highest concentration used. Mean lifespan of rotifers (n = 11, mean ± SEM) increased from 24.6±1.8 days (Control) to 58.5±3.3 days (10 µM IPAM), 81.1±3.7 days (20 µM IPAM) and 90.5±3.8 days (30 µM IPAM). To the best of our knowledge, this is the most pronounced life extension *ever* recorded in this experimental model or in similar organisms like C. elegans [11].

An unexpected finding was that IPAM treated rotifers were of larger size than rotifers *of the same age* exposed to control vehicle solution (Figure 6B,C,D), suggesting a growth promoting function for IPAM. The average rotifer length determined on day 15 of treatment (n = 10, mean ± SEM) was increased from 390 µm ±5 µm (control, vehicle treatment) to 575 µm ±6 µm (IPAM treatment). With increase in longevity, the fertility of the rotifers was also increased as measured by offspring number per parent as observed in individually housed rotifiers. In particular, the total number of viable offspring per rotifer during a lifetime rose from an average of 16 animals ±1 animal in vehicle treated control rotifers to an average of 55 animals ±1 animal after daily treatment with 30 µM IPAM in single-housed organisms (n = 10). When treated with lower doses of 10 and 20 µM IPAM, the individually housed rotifers had a total number of 52±2 and 53±2 offspring on average (Figure 6E). IPAM also increased the duration of the reproductive period per rotifer (fertile days in culture during a lifetime) to 18±1 day(s) for the IPAM treated organisms, up from 5±1 day(s) in controls (n = 10). The duration of the reproductive period per animal, as measured in the number of fertile days for each rotifer in single-housed cultures, averaged 13±1 and 16±1 day(s), when the animals were treated with the lower dose of IPAM at a concentration of 10 or 20 µM daily (Figure 6F). The effects on growth and fertility exerted by IPAM were seen at all concentrations used and were determined to be statistically significant for the treatment groups (p<0.001; n = 10).

## Discussion

In contrast to indole-3-propionic acid which bears a polar carboxyl group that is ionized at physiological pH carrying a negative charge, IPAM is non-polar and has sufficient lipophilicity (and amphiphilicity) to penetrate through biological membranes. In contrast to melatonin, IPAM is a “reversed amide” lacking the methoxy group as an aromatic substituent. Melatonin is quickly metabolized in the liver by hydroxylation in the para position to its large side chain (extensive first liver pass effect with rapid clearance) and excreted as the glucuronide or sulfate conjugate of 6-hydroxymelatonin (a pro-oxidant metabolite). IPAM, however, has a long half-life and no pro-oxidant activity (Figure 5), thus the metabolism of IPAM will be an important topic for additional investigation. Significantly, IPAM is found within the brain (Figure 1) and may be able to protect against oxidative damage, especially to mitochondria.

Severe mitochondrial dysfunction and ATP deprivation is seen in aging cells and organisms [10], [11], [14], [22]–[24]. The main sites in the mitochondrial electron transport chain at which free-radicals are produced are not fully elucidated, although at least 50% of the total free-radicals generated by mitochondria are attributed to dysfunction of complex I [25], which is in itself highly vulnerable to oxidative stress. Complex I is embedded into the inner mitochondrial membrane and serves to dehydrogenate NADH and shuttle electrons to coenzyme Q. This electron transport mechanism generates a gradient across the inner mitochondrial membrane, which provides the proton-motive force that is used in ATP synthesis. While IPA and melatonin protected against mitochondrial damage in both young (1 month) and older (20 months) rats, IPAM provided significantly greater protection (Figure 2).

To further test the effect on mitochondria, three mitochondrial toxins, including FCCP, were used to induce a collapse of the mitochondrial proton potential resulting in severe mitochondrial dysfunction and ATP deprivation as seen in senescent cells and organisms [10], [11], [14], [22]–[24]. The addition of IPAM significantly protected mitochondria function against the toxic action of all three compounds (Figure 3). IPAM was shown to have a direct effect on both the Complex I and Complex IV activities (Figure 4). Decreased activity of these complexes results in an inhibition of electron transport that is associated with higher production of ROS. Taken together, and since the activity of IPAM occurred at low nM concentrations, these data provide evidence that that IPAM can also act as a recyclable electron and proton carrier, facilitating reversible endogenous radical and redox reactions, and thereby enabling the formation of a proton gradient that drives mitochondrial ATP synthesis.

Testing the addition of IPAM to a model organism revealed astonishing results (Figure 6). Increasing concentration of IPAM resulted in survival rates reaching 300% of control, untreated rotifers. This increased survival was accompanied by dramatically increased organism size. Even though rotifers grow throughout their entire life cycle, the size increase illustrated here was independent of enhanced longevity. Further, the increased lifespan allowed for increases both in the total number of reproductive days as well a three-fold increase in the number of offspring per individual. The mechanisms involved in these processes remain to be determined.

Thus, the following conclusions can be drawn from the experiments: 1) IPAM occurs endogenously and its levels can be increased after administration of L-tryptophan; 2) IPAM has high bioavailability as it can efficiently cross the blood brain barrier and remain at high concentrations in rat brain for several hours; 3) IPAM levels cannot be increased after administration of IPA or melatonin; and 4) IPAM may impart anti-aging benefits. The latter observation may have therapeutic ramifications if one is to enhance the antioxidant defense systems in mammalian biosystems including the brain.

Similar adverse effects were observed in a prior study showing pro-oxidant activity for ascorbate (vitamin C), and trolox (a water soluble derivative of vitamin E) [1]. These findings caution against the indiscriminate use of antioxidants as cytoprotective agents which in biological systems can *increase* free-radical formation. Such effects may provide an explanation for the adverse clinical outcomes recently reported with chronic administration of substances like vitamin E [2]. We also conducted a spectrum of assays to confirm that IPAM exerted free-radical scavenging properties as compared to melatonin and other antioxidant indoles such as IPA. IPAM showed radical reducing activity which exceeded the potency of melatonin and IPA. Due to the scope of this report, these data will be presented elsewhere.

In conclusion, we report on a new and naturally occurring indole, IPAM, which showed its potential as a mitochondrial metabolism modifier and an anti-aging molecule. Likewise, IPAM may hold therapeutic potential in age-related neurodegenerative diseases and in mitochondrial disorders.
